# Tomato Polyphenol Oxidase B Is Spatially and Temporally Regulated during Development and in Response to Ethylene

**DOI:** 10.3390/molecules16010493

**Published:** 2011-01-11

**Authors:** Sally M. Newman, Piyada Tantasawat, John C. Steffens

**Affiliations:** 1 Department of Plant Breeding and Genetics, 252 Emerson Hall, Cornell University, Ithaca, NY 14853, USA; 2 Suranaree University of Technology, 111 University Ave., Muang District, Nakhon Ratchasima 30000, Thailand

**Keywords:** Differential expression, ethylene, GUS, PPO, promoter analysis, *Solanum lycopersicum*, transgenic

## Abstract

Plant polyphenol oxidases (PPOs) are ubiquitous plastid-localized enzymes. A precise analysis of PPO function in plants has been complicated by the presence of several family members with immunological cross reactivity. Previously we reported the isolation of genomic clones coding for the seven members of the tomato (*Solanum lycopersicum*) PPO family (A, A’, B, C, D, E, and F). Here we report the complex spatial and temporal expression of one of the members, PPO B. The PPO B promoter was sequenced and subjected to homology analysis. Sequence similarities were found to nucleotide sequences of genes encoding enzymes/proteins active in the following systems: phenylpropanoid biosynthesis, signal transduction and responsiveness to hormones and stresses, fruit and seed proteins/enzymes, and photosynthesis. Chimeric gene fusions were constructed linking PPO B 5′ flanking regions to the reporter gene, β-glucuronidase (GUS). The resultant transgenic plants were histochemically analyzed for GUS activity in various vegetative and reproductive tissues, and evaluated for PPO B responsiveness to ethylene induction. It was shown that PPO B expression was tissue specific, developmentally regulated, ethylene induced, and localized predominantly to mitotic or apoptotic tissues.

## 1. Introduction

Higher plant PPOs are plastid-localized oxidases that catalyze two distinct reactions: the *o*-hydroxylation of monophenols to *o*-diphenols (monophenol oxidase, tyrosinase, or cresolase activity [EC 1.14.18.1]) and the dehydrogenation of *o*-dihydroxyphenols to *o*-diquinones (catecholase or diphenol oxygen oxidoreductase activity [EC 1.10.3.2]). The quinonoid reaction products are strong electrophiles which can participate in two secondary, nonenzymatic reaction pathways: the covalent 1,4 addition of *o*-quinones to cellular nucleophiles, and the reversed disproportionation of quinones to semiquinone radicals. Semiquinone radicals can either add covalently to other molecules or generate the superoxide anion radical (O_2_^-^), which may then give rise to other reactive oxygen species (ROS [1]). These reactions can result in the formation of melanin and are responsible for an array of oxidative browning problems in post-harvest physiology of crops [2]. PPO has generated interest since its first description in 1895, and several hypotheses regarding its functions during growth and development and in response to stresses have been proposed [3,4,5]. Particularly, the defensive roles of PPO against disease and insect pests have been clearly established [5,6,7,8,9,10]. However, in most plant species studied, including tomato, multiple members of the PPO gene family have been found with varying levels of similarities in their coding and regulatory sequences, suggesting that they might perform different functions and may be mediated by distinct signal transduction systems [5]. Despite intense study, the functions of individual PPO members in plants and the factors influencing their expression are poorly understood. Promoter analysis of individual genes could provide more insight into their putative functions as well as the signal transduction pathways that mediate their expression. This knowledge will increase understanding of the limits to which PPO expression may be manipulated.

Previous studies have demonstrated that the PPO family is spatially and temporally regulated [11,12,13,14,15] and responsive to wounding and biotic and abiotic stresses as well as signaling molecules [7,16,17,18,19]. In tomato the PPO gene family consists of seven members, A, A’, B, C, D, E, and F, clustered within a 165-kb locus on chromosome 8 [20]. Among these PPO members, PPO B and E/F transcripts were the most abundant, particularly in young leaves and inflorescences [14]. Five of the seven PPOs, PPO A/A’, B, C/D, E and F, possess divergent DNA sequences in their 5’ promoter regions. Chimeric gene fusions, joining these promoter regions to the reporter gene, β-glucuronidase (GUS) were constructed and used for promoter analysis. The induced expression patterns conferred by PPO F promoter in response to injuries and pathogen infection implicated the role of PPO F in plant defense [7,18], however, the roles of other PPO members are still unclear. Here we report the specific spatial and temporal expression of one member, PPO B, in vegetative and reproductive tissues of tomato, and discuss a possible role for PPO B in apoptosis and defense.

## 2. Results and Disscussion

### 2.1. Analysis of PPO B promoter

**Figure 1:**
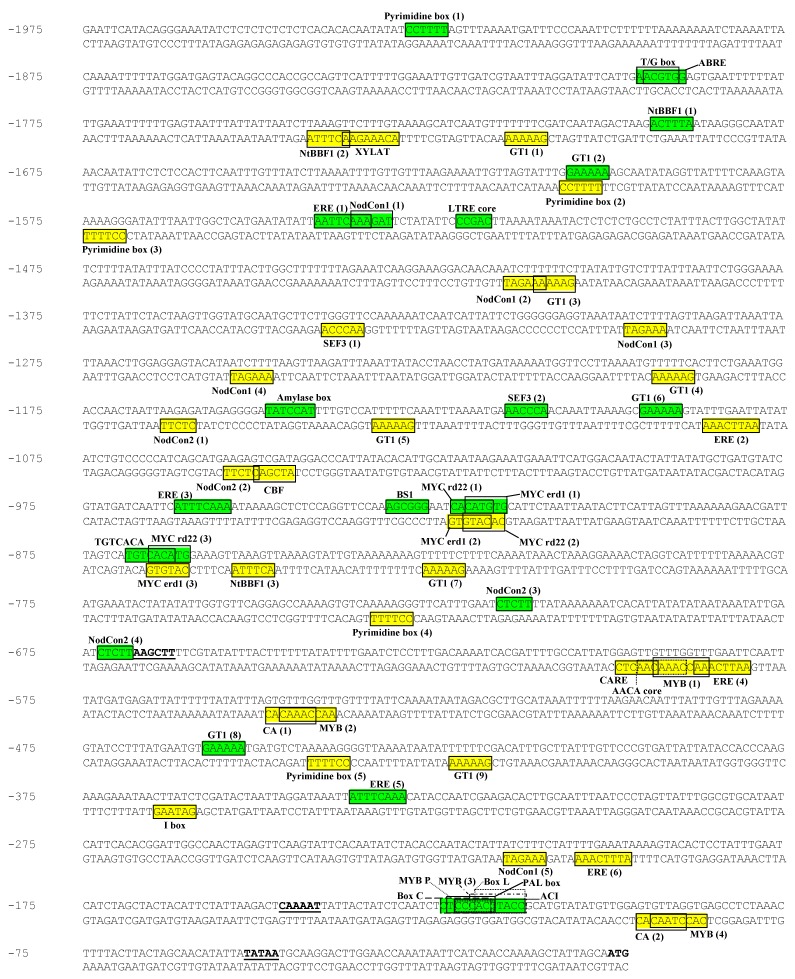
Sequenced region of the PPO B promoter showing putative *cis*-elements. PPO B bases are numbered starting with minus 1 as the first base 5′ of the ATG translational start codon. The ATG start codon is in bold. The underlined bases, 5′ to 3′ respectively, represent the 5′ terminal *Hin*dIII site of the BG plasmid construction, CAAT homology, and TATAA homology. The putative *cis*-elements are boxed.

Figure 1 shows the sequenced portion of the PPO B promoter along with the putative *cis*-acting regulatory elements from a database of plant *cis*-acting regulating DNA elements (PLACE). Most partial sequence matches (*ca.* 60-90 % homology in 30-130 bp nucleotide sequences) were primarily to the 5’ or 3’ flanking DNA sequences or introns of genes encoding enzymes/proteins which are active in one of the following systems: phenylpropanoid biosynthesis, signal transduction pathways and responsiveness to hormones and stresses, fruit and seed proteins/enzymes, and photosynthesis. These matched sequences did not necessarily contain putative *cis*-acting elements.

#### 2.1.1. Phenylpropanoid biosynthesis 

Phenolics, the substrates of PPO, are the products of the phenylpropanoid biosynthetic pathway. Moreover, the products of this pathway are also used for synthesis of lignins, phytoalexins, UV-absorbing compounds, pigments and signaling molecules, and elevated levels are usually found during wound repair and defense against pathogens [21,22]. Therefore, it is not surprising that the PPO B promoter sequence partially matched several promoters of genes in this pathway including tomato and rice phenylalanine ammonia lyase (PAL), parsley 4-coumarate:coenzyme A ligase (4CL), poplar cinnamate 4-hydroxylase (C4H), eucalyptus cinnamoyl-CoA reductase (CCR), soybean, snapdragon and *Arabidopsis* chalcone synthase (CHS), chalcone flavanone isomerase (CHI) and dihydroflavonol 4-reductase (DFR [23,24,25,26,27,28,29,30]). PAL, C4H and 4CL catalyze the first, second and last steps of the general phenylpropanoid metabolism, respectively while CCR catalyzes the first specific step in the biosynthesis of monolignols, the monomeric units of lignins [22,24]. CHS, CHI and DFR are enzymes responsible for biosynthesis of colored anthocyanins, colorless flavonols and antimicrobial phytoalexins, and their expression has been found in young petals and anthers [31]. Several genes in the phenylpropanoid biosynthetic pathway are differentially expressed and responsive to various biotic and abiotic stresses [22]. *cis*-Elements including Box P-related (MYB binding site), Box C-related, Box L-related and ACI elements are responsible for tissue-specific as well as light-regulated and/or stress-induced expression patterns of several phenylpropanoid biosynthetic genes such as PAL, 4CL, CHS, CHI, DFR and/or CCR [24,29,32,33]. The ACI element is required for vascular-specific gene expression and has been implicated in the xylem-localized regulation of gene encoding lignin biosynthetic enzymes in loblolly pine [34]. These *cis*-elements were also identified on PPO B promoter sequence (Figure 1).

#### 2.1.2. Signal transduction pathways and responsiveness to hormones and stresses 

In addition to the inducible enzymes involved in the phenylpropanoid biosynthesis, we also found partial homology between the promoter of PPO B and promoters of genes that are inducible by various plant hormones such as ethylene responsive tomato and zucchini 1-aminocyclopropane-1-carboxylate synthase (ACS), tomato E4 and tomato E8, gibberellic acid (GA) responsive barley cysteine proteinase and alpha-amylase, abscisic acid (ABA) responsive cor15a and BN115, jasmonic acid (JA) responsive tomato proteinase inhibitor II (pin2) and leucine aminopeptidase (LAP), and auxin responsive rolB [35,36,37,38,39,40,41]. ACS is the key regulatory enzyme in the biosynthetic pathway of ethylene. The expression of *Arabidopsis* ACS gene family has been reported to be differentially regulated during normal development and is induced by stresses and hormones including ethylene [42]. The seed enzymes cysteine proteinase and α-amylase are usually activated during germination to mobilize essential nutrients for seedling development. The promoters of the genes encoding these seed enzymes, a rice α-amylase, a rice cysteine proteinase and a barley proteinase, contain the CAACTC regulatory element (CARE) similar to the one found in PPO B promoter (Figure 1 [43]). Several of the genes mentioned above enable plants to survive under biotic and abiotic stresses; for example, proteinase inhibitor II has an established defensive role against insect pests, and cor15a enhances plant tolerance to low temperature and water stresses [44].

Five pyrimidine boxes (GA inducible), 1 CARE (GA inducible), one amylase box (box 1; GA inducible), 1 T/G box (JA inducible), three *Nicotiana tabacum* rolB domain B Factor 1 (NtBBF1) binding sites (auxin inducible), one low temperature responsive element (LTRE) core (ABA inducible), one ABA responsive element (ABRE; ABA inducible), and six ethylene response elements (EREs; ethylene inducible) were distributed across the PPO B promoter, suggesting that PPO B might be regulated by these hormones (Figure 1 [40,41,43,45,46]). The GA inducible pyrimidine boxes and box 1 element were previously reported in the promoters of two pineapple PPO genes, *PINPPO1* and *PINPPO2*, and may be involved in cold response [47]. Moreover, pathogen and salt induced GT1, cold and drought induced LTRE core, and dehydration responsive C box binding factor (CBF), ABRE and MYC elements were also observed in the PPO B promoter sequence (Figure 1 [45,48,49,50]). Patatin and heat shock proteins both respond to stress along with PAL and 4CL in higher plants. Patatin transcripts decrease while heat shock protein transcripts increase [51]. Partial homologies to promoters of potato patatin II gene, and soybean 17.5-M and 18.5-C heat shock protein genes overlapped in the PPO B upstream region [52,53].

Pathogen-related (PR) proteins are low molecular weight proteins which accumulate systemically in association with the hypersensitive reaction. Identified PR proteins include chitinases and β-1,3-glucanases. Chitinase may directly inhibit fungal growth and/or release defense-related elicitors from fungal cell walls whereas β-1,3-glucanase may help degrade fungal cell wall in addition to its involvement in normal plant development such as seed germination, growth, and flower development [54]. The 5’ flanking region of PPO B partially matched peanut chitinase II gene intron 1, and tobacco and barley 1,3 β-glucanase 5′ flanking regions [55,56,57]. Also located at the same upstream site was a partial match to potato proteinase inhibitor I gene 3′ flanking region [58]. Partial homology was also found with the 3′ flanking sequence of horseradish peroxidase (PO) gene [59]. These enzymes have been implicated in plant defense against pathogens and insect pests. In addition, PPO B 5’ flanking sequence also contained partial homology to sequence of a gene encoding Sn-2, a fruit-specific wound-stimulated protein in bell pepper [60].

The G protein family of transmembrane signaling molecules regulates the stimulation of cAMP synthesis by hormones. Components of this signaling system, including cyclic nucleotide phosphodiesterase, ras, rap, tuf, CA^2+^-binding and ion channel genes, have been identified in various organisms [61]. cAMP may have a possible role in stress signaling in plants via the regulation of K^+^ and Ca^2+^ channels that lead to extracellular alkalization, affecting the potentiation of the oxidative burst leading to defense gene activation [62]. PPO B promoter had partial sequence matches to flanking regions of cAMP responsive genes and to the components of the cAMP signaling system such as *Dictyostelium* cAMP receptor (CAR1), *Dictyostelium* CRAC (a coupling protein that acts to connect free G protein subunits to adenylyl cyclase activation), *Dictyostelium* cyclic nucleotide phosphodiesterase, *Dictyostelium* rap 1, *Dictyostelium* rasG, *Astasia longa* tufA and *Tetrahymena* calcium-binding protein (TCBP-23) [63,64,65,66,67,68,69]. An ABRE-related sequence identified in the upstream regions of 162 Ca^2+^-responsive upregulated genes that function in dehydration induction and dark-induced senescence was observed in the PPO B promoter (Figure 1 [49,70].

Plants also maintain intricate interactions with symbiotic microorganisms. The *Rhizobium*-legume interaction leads to symbiotic nitrogen fixation carried out by differentiated bacteria within the root nodule [71]. Interestingly, the 5′ flanking regions of several genes involved in root nodulation were partially homologous to that of PPO B, e.g., soybean nodulin 22, *Sesbania rostrata* leghemoglobin [72,73]. The PPO B promoter also partially matched a 5′ flanking region of tobacco and barley nitrate reductase genes [74,75]. Both of the putative nodulin consensus sequences, NodCon1 and NodCon2, which confer nodule specificity in nodulin genes, appeared on the PPO B promoter region (Figure 1 [72]).

#### 2.1.3. Fruit and seed proteins/enzymes 

Polygalacturonase (PG) is the primary determinant of cell wall polyuronide degradation during fruit ripening, organ abscission and pod dehiscence. PG plays important roles on seed germination, cell expansion, pollen grain maturation, anther dehiscence, abscission, fruit ripening, and pod shatter. Its expression was apparent at the dehiscence zone of pods, at the sites of leaf, flower and seed abscission and at pollen grains [76,77,78,79]. PPO B was found to have partial homology to the tomato PG C, D, E and/or F elements at similar spacing relative to the ATG translational start [80,81]. Interestingly, the expression of PPO B in abscission zones, the basal cell of trichomes, pollen grains and pistils was consistent with that of *Arabidopsis* and tomato PGs [79,82].

PPO B promoter has partial homology to that of rice ADPglucose pyrophosphorylase (AGPP) which catalyzes the key regulatory step in starch biosynthesis and maize UDP-glucose starch glycosyl transferase [83,84]. The expression of AGPP in rice seeds was found to be endosperm-specific [84]. The AACA core element that has been shown to confer endosperm specific expression in rice glutelin genes and soybean embryo factor 3 (SEF3) element which was observed in seed storage protein β-conglycinin (7S globulin) gene were found in the PPO B 5’ flanking region (Figure 1 [85,86]). In addition, the (CA)n element that is required for embryo and endosperm-specific expression was also observed (Figure 1 [87]). PPO B promoter also contained *cis*-element ACGT core sequences that were found in promoter regions of several seed-expressed genes and PAL [88,89].

#### 2.1.4. Photosynthesis

5′-Flanking regions of wheat chloroplast PsaC gene for photosystem I 8 kDa subunit, barley chloroplast PsaC photosystem I subunit C, pea ribulose bisphosphate carboxylase small subunit (RBCS) 3A, pine, wheat and maize chlorophyll a/b binding proteins (CABs) had partial homology to that of PPO B [90,91,92,93,94]. The I box previously found in tomato and *Arabidopsis* RBCS promoter regions, which seems to be involved in light-regulated and/or circadian clock-regulated gene expression of photosynthetic genes, was observed in the PPO B promoter (Figure 1 [95]).

Several putative *cis*-elements directing tissue-specific expression of PPO B were identified, including the pollen-specific Pollen1 element, the embryo and endosperm-specific (CA)n element, the vascular-specific ACI element, the xylem-specific XYLAT element, and the fruit-specific TGTCACA motif enhancer element (Figure 1 [87,88,96,97]). In addition, nine GT1 elements were observed in the 5’ flanking region of PPO B (Figure 1). GT elements, possessing both a positive and a negative function in modulating cell type-specific transcription, have been found in the promoter region of several genes encoding diverse functions such as RBCS, CAB, ATP synthase, phytochrome A, CHS, PR protein, alcohol dehydrogenase and pollen-specific LAT52 [98].

### 2.2. Spatial and temporal expression conferred by the PPO B promoter

**Figure 2:**
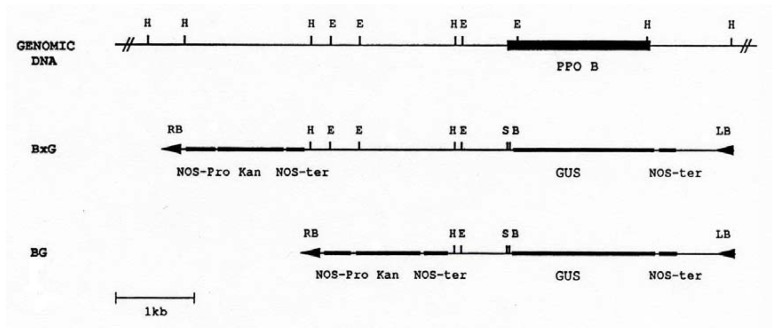
Restriction map of the PPO B promoter region and chimeric PPO B:GUS reporter constructions. The coding region of PPO B is indicated as a solid box. Construct BxG contained 2.52 kb PPO B 5′ flanking DNA (bases -2520 to -1). Construct BG contained 0.67 kb (bases -668 to -1). The PPO fragments were inserted into the promoterless GUS cassette binary cloning vector PBI101.3, and were used to transform tomato cotyledons via *Agrobacterium* mediated transformation. E, *Eco*RI); H, *Hin*dIII; S, *Sma*I; B, *Bam*HI; GUS, β-glucuronidase; Kan, kanamycin; LB, left border; RB, right border; NOS-ter, nopaline synthase terminator; NOS-pro, nopaline synthase promoter.

PAL and 4CL are key enzymes in the shikimic acid pathway. Since the substrates of PPO are phenolics, the end products of this pathway, it seemed likely that PPO B′s large region of homology (60-70%), -878 to -759, to the promoters of genes encoding these two enzymes could represent a control region over PPO B expression. To test this hypothesis, the two PPO B promoter:Gus fusions shown in Figure 2 were constructed. BxG contained 2.52 kb of 5’ flanking sequence, whereas, construct BG took advantage of a *Hin*dIII site, 107 bp 3’ of the PAL/4CL region, to delete the upstream sequences, leaving only 0.67 kb of 5’flanking sequence. These two constructs were transformed into tomato to generate PPO B:GUS fusion transgenic plants for histochemical GUS analysis.

**Figure 3:**
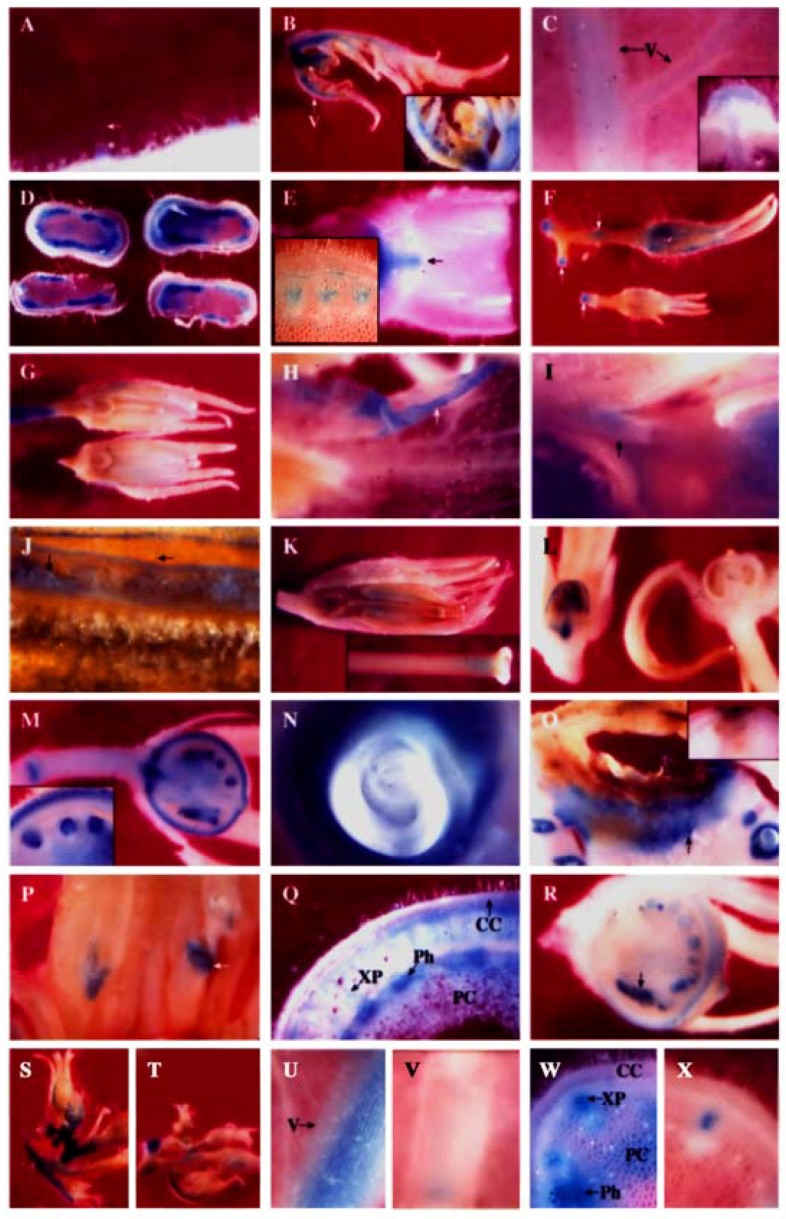
Histochemical staining of β-glucuronidase activities in transgenic PPO B:GUS fusions (at 8× or 40× magnification). A, Type I trichome (arrow); B, Apical leaf. The inset shows young flower buds; C, Apical leaflet at node 4. The inset shows cross-section; D, Stem at internode 4; E, Stem at node 8. Axillary bud (arrow); F, Flowers (3-8 mm); G, Flowers (10 mm); H to J, Anthesis flowers; H, Petal; I, Anther attachment; J, Pollen; K, Post-anthesis flower. The inset shows junction of stigma and style; L, Two fruits (3 mm) collected on the same day from the same plant; M, Fruit (5 mm); N, Green fruit developing ovule; O, Green fruit with blossom end rot. The inset shows no staining in green fruit with blossom end rot of transgenic PPO E:GUS fusion; P, Anthesis flower with stunted anthers (arrow); Q, Stem at internode 8 with elevated expression; R, Fruit (5 mm) with both normally developing ovules and stunted ovules (arrow); S, U and W, Induced PPO B in response to ethylene. T, V and X, PPO B expression in control plants. S and T, Apical leaves and flower buds; U and V, Apical leaflet at node 7; W and X, Stem at internodes 7 and 8. CC, cortical cell; PC, pith cell; Ph, phloem; XP, xylem parenchyma; V, vein.

Figure 3 shows histochemical GUS activities of the two PPO B:GUS fusions, BxG and BG. Deleting 1.33 kb of 5′ flanking sequence from the BxG construct made no difference in the observed GUS activity patterns, indicating that the PAL/4CL promoter region is not required for steady state expression of PPO B. Similarly, the pattern of GUS expression in independent transgenic lines was conserved although the levels of staining varied slightly among individual plants.

It was found that PPO B was differentially expressed during growth and differentiation in both vegetative and reproductive tissues. In most organs, PPO B expression was found to be highest in young tissues and decreased with age, and in most tissues, expression was strongest in vascular tissues and abscission zones. Expression was frequently found in patches in a given tissue. This individual cell expression is typical of enzymes in general phenylpropanoid metabolism [99]. All tissues showed staining of type I trichome bases (Figure 3A) and occasionally type IV trichomes (data not shown).

#### 2.2.1. PPO B expression patterns in leaves

All epidermal and internal tissues of petioles leading to leaflets and floral buds were stained. The major veins of the apical leaves showed some epidermal but predominantly internal tissue expression (Figure 3B). Some staining of leaflet tissues was seen but to a much less extent. The PPO B expression levels in all leaf tissues subsequently declined in older leaves. Apical leaflets at nodes 4 and 8 showed only light staining of major vein vascular and cortical tissues. Node 8 expression was reduced relative to node 4 (Figure 3C, data not shown).

#### 2.2.2. PPO B expression patterns in stems

Similar temporal PPO B expression was also found in stems. Stems at internode and node 4 exhibited intense GUS activities in the differentiating xylem, xylem parenchyma and pith tissues, particularly in the cells between the vascular bundles. Cortex, cambium, and phloem tissues expressed, but less intensely and more sporadically (Figure 3D). Expression was also noted in the vascular traces and the axillary buds (data not shown). However, by the internode and node 8 stage, expression had diminished in stem tissues, yet young axillary bud tissues were seen to be stained intensely (Figure 3E). Staining was also observed in node 8 abscission zones.

#### 2.2.3. PPO B expression patterns in roots

PPO B promoter-driven GUS expression was not detected in roots, whether the root samples were soil grown lateral roots or vermiculite grown adventitious roots from cuttings (data not shown).

#### 2.2.4. PPO B expression patterns in flowers

Apical tissue showed intense staining of most tissues associated with young flower buds (Figure 3B inset). Teasing back the sepals revealed the developing ovary tissues to be darkly stained (not shown). Flowers (3-8 mm) showed staining of sepals, basal ovary tissues, pedicels, and pedicel joints (Figure 3F, data not shown). As the flowers developed, staining was lost from the ovary tissues, but appeared in anther and stigma (Figure 3G). At anthesis, the flowers showed GUS staining of sepals, petals, pollen, ovary attachment area, anther attachment area, petal attachment area, pedicels and pedicel joints (Figures 3H, I, J, data not shown). After anthesis PPO B expression remained in the pollen, petal attachment area, ovary attachment area, and pedicel joints. In addition, GUS staining was also observed in stigma/style junction, style/ovary junction and internal style tissues (Figures 3K, data not shown). The expression in the internal style tissues may stem from the germinating pollen tubes where PPO B transcripts were found [14].

#### 2.2.5. PPO B expression patterns in fruits

As the ovary tissues began to swell during fruit formation, staining of the ovary tissues began again. The timing of this second round of ovary tissue expression varied. Figure 3L shows two 3 mm fruits picked from the same plant on the same day, and processed identically. One was expressing fully, the other had not begun. Usually expression began at the 4-6 mm stage. Once the expression began, all areas of the developing fruits that expressed did so simultaneously. The outer half of pericarp (an expression pattern also noted for the PG gene [80]), developing seed coats of ovules, vascular tissues, placental tissues and pedicel joints all stained clearly (Figure 3M). In addition, the ovary attachment area and petal/anther attachment area which were seen to stain lightly in anthesis flowers were now intensely stained (Figures 3I,M, data not shown). The single layer of surface placental cells surrounding ovules also expressed as well as the endosperm tissues within the ovules (Figure 3M). The patterns and intensity of GUS staining of fruit tissues did not change between the 5 mm and full-sized green fruit stages. Figure 3N showed that as ovules progressed through seed development, the endosperm and developing seed coat remained intensely stained while the developing embryo was expressionless.

#### 2.2.6. Abnormal PPO B expression patterns

Several interesting samples were found during the histochemical analysis which may indicate the inducibility of PPO B expression. Figure 3O shows the fruit of BxG plants which developed blossom end rot, a condition brought on by calcium deficiency. Here PPO B promoter driven GUS expression formed apparent lines of defense at the site of infection. Figure 3P shows intense GUS staining of anthers stunted in development at anthesis. Normal anthers in the background showed no staining. Figure 3Q shows atypical stem at internode 8 expression. Normally, PPO B promoter driven GUS expression in stem tissues was diminishing and restricted to only vascular tissues by node 8. However, two stems out of 39 examined had elevated expression levels, suggesting that an induction event had occurred. This pattern of induction is different from that induced by *Pseudomonas syringae* [7], but similar to the one induced by ethylene (Figure 3W). Figure 3R shows a final example of putative PPO B promoter induction, a 5 mm fruit which contained both normal ovules and ovules arrested in development. The stunted ovules stained much more intensely for GUS activities than did the normal ovules.

### 2.3. Ethylene Induction of PPO B Promoter

**Figure 4:**
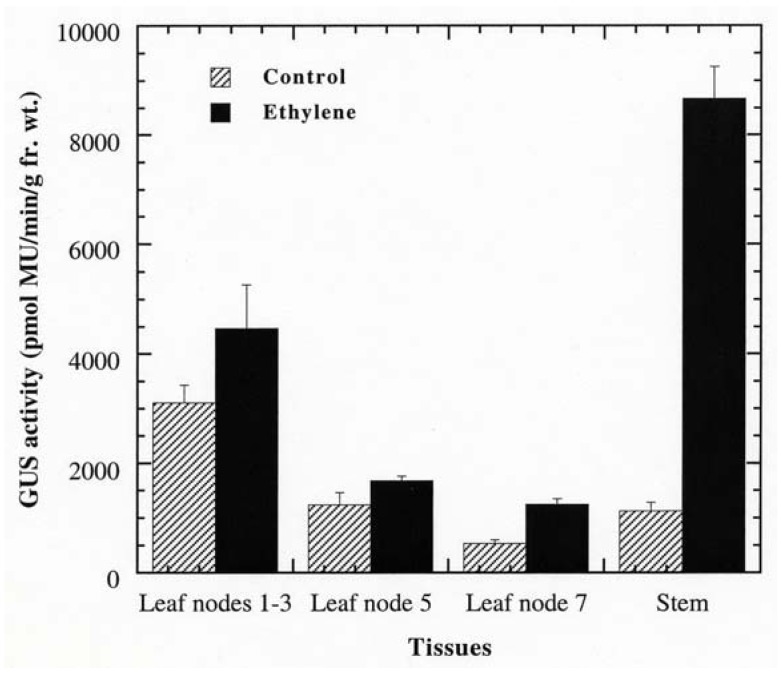
Responsiveness of PPO B to ethylene. BxG shoots were incubated in 1 mM ethephon in NaPO4 buffer, or in NaPO4 buffer alone (control). After 48 h, apical leaf nodes 1 to 3, leaf nodes 5 and 7, and the stems at internodes 7 and 8 were collected for fluorometric GUS assay. Fr. wt., fresh weight; MU, methylumbelliferone.

Promoter analysis revealed that PPO B shared extensive homology to the promoters of the tomato ethylene related genes: ACS, the key regulatory enzyme in the biosynthetic pathway of ethylene; Le E8, an ethylene-responsive fruit ripening gene; and PG, the fruit wall softening gene which increases as ethylene increases during fruit ripening. Six EREs, which have been shown to mediate ethylene-induced activation, were found along the PPO B promoter sequence. In addition to sequence homology, PPO B driven GUS expression was intense in tissues typically associated with elevated ethylene levels: rotting fruit (blossom end rot; Figure 3O), stunted anthers (Figure 3P), and stunted ovules (Figure 3R). It seemed likely that PPO B expression might be regulated by ethylene. To test this hypothesis, we evaluated PPO B-driven GUS expression levels in control and ethylene-treated tomato shoots. BG and BxG shoots were incubated in solution of 1 mM ethephon in 0.1 M NaPO_4_ pH 7.0, or 0.1 M NaPO_4_ alone as a control for 48 h. After 48 h, GUS activities of leaves at nodes 1 to 3, 5, 7, and stems at internodes 7 and 8 were evaluated qualitatively and quantitatively. Ethylene induced 1.4, 1.4, 2.3, and 7.7 fold increases in PPO B promoter driven GUS expression in leaves at nodes 1 to 3, 5, 7, and stems, respectively (Figure 4). While GUS activities of leaf nodes 1 to 3 and 5 were not significantly different from those of control (*P* > 0.05), leaf node 7 and stems exhibited significant increases in GUS activities (*P* < 0.05). pBI121 and nontransformed controls did not show increases in GUS expression upon ethylene induction.

Histochemical GUS assay showed that expression patterns of BG and BxG controls in all tissues examined were similar, but with slightly different expression levels. The control expression pattern was as described above but to a lesser extent since less incubation time was used. Apical leaves showed expression in the major veins (Figure 3T). All cell types in petioles leading to leaflets and buds were stained. The only expression found in young flower buds was in sepal (Figure 3T) and ovary tissues (data not shown). After ethylene treatment, PPO B-driven GUS expression was induced to slightly higher levels in tissues possessing the GUS activity under normal development (Figure 3S). In addition, low levels of expression were also found in petals and leaf mesophylls. In some sections, induced GUS expression was also evident in leaf epidermis and all types of trichomes (data not shown).

Ethylene induced relatively higher levels of PPO B-driven GUS expression in major vein vascular and cortical tissues, and in petiole xylem parenchyma and pith cells of apical leaflets at both nodes 5 and 7 (Figures 3U,V, data not shown). Similar to apical leaf nodes 1 to 3, additional induced GUS expression was also observed in leaf mesophylls, epidermis and all types of trichomes of some sections (data not shown).

Stems exhibited the highest responsiveness of PPO B to ethylene (Figure 3W). While control plants showed minimal GUS expression in only vascular parenchyma, substantial induced PPO B-driven GUS expression was also found in pith cells (Figure 3W,X). In addition, induced GUS expression of some sections could also be observed in epidermis, cortex, and phloem cells (Figure 3W).

### 2.4. Discussion

#### 2.4.1. Spatial and temporal expression of PPO B under normal growth and differentiation

Polyphenol oxidases utilize phenolic end products from the phenylpropanoid biosynthetic pathway as substrates for oxidation. Like the enzymes involved in general phenylpropanoid metabolism, PPOs are present in plants under normal conditions, and reported to be inducible following wounding, pathogen attack, and ethylene application [5,7,16,17,18,22]. We have previously reported the co-localization of PPO and phenolics in developing fruits, type VI trichomes, young leaves, styles and root tips of tomato [14]. Extensive homology between promoter sequences of PPO B (-878 to -759) and PAL and 4CL suggested a possible co-ordinate regulation of these genes to sustain optimal levels of PPO and its substrates, which may be vital for plants under normal growth and differentiation and stress conditions. The significance of this region was tested using two chimeric gene fusions, joining the PPO B promoter region to the reporter gene, GUS. One construct, BxG, contained the PAL/4CL region (-878 to -759), the other, BG, did not. Introduction of these constructs into tomato plants and subsequent histochemical analysis of GUS expression patterns revealed no significant differences in expression patterns or intensities between BxG and BG plants, indicating that, under normal growth conditions, PPO B expression is governed by 0.67 kb or less of 5′ flanking sequence and that the upstream PAL/4CL region is not required for this expression. The *cis*-regulatory sequences controlling these expression patterns appeared to reside largely within the first 0.67 kb of the PPO B promoter. It is interesting to note that the shorter promoter of BG plants still contained several of the *cis*-elements which were found in PAL’s promoter including Box C-related, PAL Box, Box L-related and ACI elements localized at the -124 to -113 site (relative to the translational start) as well as the Box P-related (MYB binding site) element (Figure 1), which might be sufficient to direct the observed expression patterns. Functional analysis of 5’ deletions and selective mutations will be required to assess the possible regulating roles of these *cis*-elements.

The preferential expression of PPO B in vascular tissues of all organs observed here was similar to the expression of PAL, 4CL and CCR, which all possess ACI elements in their promoters. Note that the BS1 element which specified vascular expression in CCR gene was also found in the PPO B promoter [29]. These common motifs may provide a mechanism by which different steps of phenylpropanoid metabolism and PPO are co-ordinately regulated. The co-ordinate regulation of PPO and phenylpropanoid biosynthesis underscores the importance of PPO in plant defense. PPO may act after the barriers provided by the induced phenylpropanoid pathway, e.g. phytoalexins and lignification, are overcome. Once plant cells are disrupted, PPO activity diverts phenolics to quinone production, aiding cell death and providing additional polymerized phenolic barriers to secondary infection [18].

Under normal greenhouse conditions, PPO B expression was found to be tissue-specific and developmentally regulated. PPO B promoter-driven GUS expression generally appeared to be localized to young mitotic tissues and to tissues which undergo apoptosis or programmed cell death. PPO B expression was found in rapidly dividing tissues of young flower and leaf buds, young ovaries, fruit tissues, and seed endosperm. Strong PPO B expression was also found in xylem parenchymal cells. These cells form tyloses in adjacent xylem tracheary elements during abscission, pathogen attack, and mechanical injury [100]. During differentiation into xylem tracheid cells, xylem vessels developing from cambium cells undergo autolysis [101]. The XYLAT element identified among the promoters of the genes regulating secondary xylem development was identified in the PPO B promoter [88].

PPO B expression was found in anther and pollen materials. Nine Pollen1, one of the two co-dependent regulatory elements responsible for pollen specific activation of tomato LAT52 gene encoding a cysteine-rich protein, were found to be distributed across the PPO B promoter region [96]. In addition, PPO B expression was found at the base of all structures which normally abcise (a process involving apoptosis) during development: petals, sepals, stigmas, and fruits, and leaf and pedicel abscission zones. During abscission of plant organs, cell wall degradation is associated with an increase in the activity of several hydrolytic enzymes including β-1,4-glucanases, PG and other proteins such as expansins, which may either contribute to the cell separation process or play a role in protecting the exposed surface from pathogen attack. In oilseed rape and/or *Arabidopsis*, expression of PG was also observed in the abscission zones at the base of the anther filaments, petals and sepals, the dehiscence zone of anthers and siliques, and pollen grains [78,79]. In addition to the role of PG in cell separation, Hong *et al*. [82] proposed that PG may play a secondary role in abscission zones and pistils by releasing oligogalacturonides from cell walls to mount a local defense response. The presence of PPO B in these senesced tissues may not necessarily implicate a causal role for PPO in the cell death process, but may reflect the defensive roles of PPO at wound sites to discourage secondary pathogen colonization.

In addition, programmed cell death is thought to be involved in the hypersensitive response (HR) of plants to pathogens [102,103,104,105]. Although PPO has been implicated in pathogen response and the PPO B 5′ flanking region showed sequence similarity to similar regions of PR proteins and inducible phenylpropanoid biosynthetic enzymes, our previous report showed that PPO F but not PPO B was induced in leaf cells at the periphery of *Pseudomonas syringae* developing lesions and as a result of HR [7]. These results suggest that PPO B is unlikely to play a role in HR cell death, and that individual PPO gene family members are under elaborate controls to differentially express in response to specific developmental and environmental cues. This further implicates the differential roles conferred by individual PPO gene members. PPO F, which is inducible by bacterial and fungal infection as well as mechanical wounding, and is responsive to JA, SA and ethylene, was proposed to be important to general resistance [18]. In contrast, PPO B, the expression of which was minimally induced upon pathogen infection, but substantially increased under water stress, may play a distinct role [19].

#### 2.4.2. Responsiveness of PPO B to ethylene

Promoter analysis also revealed extensive sequence homology to the 5′ flanking regions of the tomato ethylene related proteins ACS and Le E8. Although PG is not induced by ethylene, the synthesis of PG and ethylene are temporally coordinated [81]. Ethylene synthesis increases during several stages of plant growth and development, seed germination, abscission, leaf and flower senescence, and fruit ripening. It is induced by a variety of external factors, including wounding, pathogen attack, elicitor treatment, cold injury, and drought [44]. Several enzymes including PPOs have been shown to be induced in response to ethylene [106]. In tomato PPO activity increased significantly in ethylene-treated excised stem segments [107]. In addition to sequence homology, PPO B-driven GUS expression was also intense in tissues typically associated with elevated ethylene levels. The ethylene responsiveness of the PPO B promoter was tested by treating BxG and BG shoots with 1 mM ethephon. Although no statistical differences were found between ethylene-treated and control BxG plants at apical leaf nodes 1 to 3 and leaf node 5, a trend showing relatively higher level of expression in tissues normally expressing PPO B was observed qualitatively. In leaf node 7 and stem tissues where significant increases in BxG GUS activities were detected upon ethylene treatment, a substantial increase in GUS activities was observed qualitatively in tissues normally expressing PPO B. In addition, induced GUS activity was also found in stem pith cells. And in some sections, induced PPO B-driven GUS expression was also evident in leaf mesophylls, epidermis, all types of trichomes, and stem epidermis, cortical, and phloem cells. A comparable induction pattern was observed between BxG and BG plants, suggesting that *cis*-element(s) residing in the 0.67 kb flanking sequence of the BG construct were sufficient to confer ethylene responsiveness to the GUS gene. It is interesting to note that only three of the six ERE elements were found in the 0.67 kb flanking sequence of BG, but they seem to be sufficient to confer the ethylene-induced expression patterns in both leaves and stems, suggesting the redundancy of the ERE element in the PPO B promoter.

Ethylene responsiveness of PPO B appears to be tissue- and developmental stage-specific. The nonsignificant induction of PPO B promoter activity in leaf nodes 1 to 3 and 5 could explain the inability to detect increased PPO B mRNA levels in these tissues upon mechanical wounding and *A. solani* infection [18]. The differential induction pattern is not uncommon for defensive genes. The class III glycine-rich protein is preferentially induced to higher levels in stems as compared to leaves in response to wounding [108]. In contrast, the viroid-induced increase in proteinase P69 is highest in the mature leaves relative to younger and older leaves, stems, and roots [109]. Another member of the PPO gene family, PPO F, was also responsive to ethylene treatment, but with different activation levels and patterns. While PPO B was significantly induced in older leaves (node 7; 2.3-fold) and stems (7.7-fold), PPO F was significantly induced only in older leaves (2.6-fold). The ethylene-induced PPO F expression in older leaves, however, occurred predominantly in leaf veins, similar to PPO B [18].

#### 2.4.3. Comparison of PPO B promoter activities and PPO B transcript accumulation

PPO B promoter is active in a developmentally specific and tissue-specific manner. The differential accumulation of PPO transcripts was previously reported by several authors [5]. Shahar *et al*. [11] reported recovering only PPO F clones from a floral tissue cDNA library, but the PPO B:GUS fusion experiments demonstrate that the PPO B promoter is also active in floral tissues. Concurrent with the GUS expression studies, *in situ* hybridization locating PPO B mRNA transcripts in the same floral tissues were reported by Thipyapong *et al*. [14]. In other tissues, where similar stages were examined, the GUS and the *in situ* hybridization results were generally in good agreement, suggesting that PPO B is at least in part transcriptionally regulated. The accumulation of PPO B transcripts in ovules of developing fruits and phloem cells of stems paralleled PPO B promoter activities. However, PPO B promoter activities were observed in other fruit and stem tissues in which PPO B transcripts were not detectable. In addition, a few more discrepancies were found between PPO B promoter driven GUS activation and PPO B transcript accumulation.

In mature leaf (node 5) mesophylls, *in situ* studies found transcripts whereas GUS activity was confined to the major veins. However, GUS activity was found in mature leaf mesophyll cells after ethylene induction (discussed above). *In situ* hybridization did not find PPO B transcripts in type I and IV trichomes. However the GUS expression in these cells was also found to be very sporadic; therefore, a positive trichome could easily have been missed in *in situ* thin sectioning. The GUS activities in mature pollen grains when the PPO B transcripts were no longer detected may be the remnant of earlier expression in microspore mother cells. Lastly, *in situ* studies found B transcripts in root apical meristem, whereas no GUS activity was found in roots [14]. This difference could indicate the need for 3′ flanking sequences or additional 5′ flanking sequences further upstream to correctly direct the expression of GUS in the roots.

#### 2.4.4. Putative roles of PPO B

Taken together, the tissue localization and promoter sequence homologies suggest a role for PPO B in programmed cell death during normal growth and differentiation, and under conditions of elevated ethylene levels. Plant developmental stage is critical in perception and/or competence to respond to ethylene. The responsiveness of PPO B to ethylene in older leaf and stem tissues may be essential to normal senescence processes in plants. Ethylene also induced PPO B expression in leaf abscission zones where Hagemann [110] reported increases in phenoloxidase activity during differentiation. It was found that drought stress induced similar activation of PPO B in abscission zones, stem and leaf tissues [19], suggesting that ethylene might be a primary effector of these responses under conditions of water stress. Six ERE elements were found in the PPO B 5’ flanking region, substantiating the involvement of ethylene mediated regulation. It was found that the promoters of various genes that are ethylene-inducible contain EREs. The expression of one of these genes, *SmCp* encoding a cysteine proteinase, which has 3 ERE elements in its promoter also coincides with the developmental events leading to programmed cell death [46]. In addition, GA was also shown to promote programmed cell death and increased membrane rupture and vacuolation in barley [111]. Several GA inducible *cis*-elements were found in the promoter of PPO B as well as the promoters of two pineapple PPO genes [47].

PPO′s properties make it a good candidate for an apoptotic role. Normally PPO is sequestered in the chloroplast away from its phenolic substrates in the vacuole. Upon cell rupture or degradation PPO would be released, allowing the enzyme to come in contact with its substrates and generate quinones. Quinones and the ROS generated could be toxic to pathogens and plant cells simultaneously. Additionally, the high accumulation of PPO B in abscission zones or senesced tissues would serve to promote cell death and to prevent pathogen egression into exposed tissues once the tissues fall off. The variability of the quinone secondary reaction products would make resistance development difficult. However, specific inhibition of PPO B by RNAi technology might be required to elucidate its functions more clearly.

## 3. Experimental 

### 3.1. Sequencing and comparative analysis of PPO B promoter sequence

PPO B 5′ flanking genomic fragments derived from a genomic library of VFNT cherry tomato DNA [20] were ligated into the pBluescript SK- cloning vector (Stratagene, La Jolla, CA, USA). DNA sequencing was performed via a T7 polymerase microtiter plate DNA sequencing system (Amersham, Piscataway, NJ). The sequence of the PPO B promoter was retrieved from annotated non-redundant collection of the Eukaryotic Promoter Database (http://www.isrec.isb-sib.ch/ssa/oprof.html) under accession number Z12834, and homologically searched against non-redundant nucleotide library (http://blast.ncbi.nlm.nih.gov/Blast). A sequence with a translational start site and its upstream region covering motifs TATA, CAAT or GC box, was mapped to the double stranded PPO B promoter using Sequence Extractor (http://www.bioinformatics.org/ seqext/). In addition, putative *cis*-acting elements in the promoter DNA sequences were identified by Web Signal Scan Program using a database of plant *cis*-acting regulatory DNA elements (PLACE; http://www.dna.affrc.go.jp/PLACE/signalscan. html).

### 3.2. PPO B promoter:GUS fusion constructs

To create the GUS fusion construct BG, a pBluescript clone containing a 2.5 kb *Hin*dIII fragment carrying both 5′ flanking and coding PPO B DNA was subjected to polymerase chain reaction (PCR) using oligonucleotide primers directed at the first 15 bases of the minus strand 5′ of the ATG translational start codon, and the universal primer M13 reverse present upstream of the multicloning site in pBluescript. The resulting 0.67 kb 5′ flanking DNA PCR product was digested with the restriction endonuclease *Eco*RV, to remove the M13 reverse primer region, then ligated into the *Sma*I site of pBluescript using standard methods [112]. The 0.67 kb fragment was removed from pBluescript with a *Hin*dIII and *Bam*HI endonuclease restriction digestion and ligated into the promoterless GUS cassette binary cloning vector PBI101.3 [113] cleaved with *Hin*dIII and *Bam*HI. To create BxG, the BG clone was digested with *Hin*dIII, as was a second pBluescript clone carrying an additional 1.85 kb of 5′ flanking DNA. The pBluescript fragment was ligated into BG, thereby creating BxG, a PPO B:GUS fusion construct possessing 2.52 kb of PPO B 5′ flanking DNA.

### 3.3. Tomato transformation

Chimeric BG, BxG, pBI101.3 (promoterless vector control), or pBI121 (CAMV 35S promoter driven constitutive GUS expression [113]) constructs were electroporated into disarmed *Agrobacterium tumefaciens* strain LBA 4404 [114], and used to transform tomato (*Solanum lycopersicum* cv Money Maker) cotyledons by the method of Frary and Earle [115]. Correctly transformed plants were identified by Southern blot analysis using the GUS gene coding region as the radioactively labeled hybridization probe. At least two independent transgenic lines of each of the BG and BxG GUS fusions were used for histochemical GUS analysis.

### 3.4. Plant materials

Tomato plants were grown under standard greenhouse conditions with a 16 hr photoperiod until 2-4 months of age. The following hand sections were removed from the plants for histochemical GUS activity determination: apex, stems at internode and node 4, apical leaflet at node 4, stems at internode and node 8, apical leaflet at node 8, immature flowers from longitudinal size range 2-11 mm, flowers at anthesis, post-anthesis flowers, fruits at the 3-6 mm diameter size, green fruit at full size but prior to reddening, lateral roots grown in soil, and adventitious roots from cut stems started in vermiculite. The 4-6 mm fruits were selected based on the finding of Gillaspy *et al*. [116], fruits of this size in VFNT cherry tomato represented an intermediate stage in fruit development where locular tissue had differentiated away from the placental tissue, but the fruit had not yet reached full size. Full-sized, red fruits were initially collected, but on finding that endogenous factors in the red fruit turned nontransformed plant sections blue, this stage was no longer examined, and full-sized green fruits were substituted.

### 3.5. Histochemical localization of β-Glucuronidase activity

The plant sections described above were immersed in GUS buffer (10 mM EDTA pH 8.0, 10 mM NaPO_4_ pH 6.8, 0.0025 mM potassium ferrocyanide, 0.001% Triton X-100, 10 mM diethyldithiocarbamic acid, and 0.5 mg/mL 5-bromo-4-chloro-3-indolyl β-glucuronide [X-Gluc; Jersey Lab Supply, Livingston, NJ, USA]). The buffer was a modification of McCabe *et al*. [117]. Samples were vacuum infiltrated, then placed at 37 °C for 22 h. Chlorophyll was then extracted from the tissue with successive 95% ethanol washes. Samples were stored in 70% ethanol until observation under a dissecting microscope equipped with a camera.

### 3.6. Ethylene induction of PPO B expression

BG and BxG shoots containing apical leaf nodes through node 8 leaves were cut and put in a solution of 1 mM ethephon, an ethylene releasing compound, in 0.1 M NaPO_4_, pH 7.0, or in a solution of 0.1 M NaPO_4_ alone as a control. Tomato shoots were then incubated in glass jars for 48 h, and four replications were used for each treatment. In addition, pBI121 and nontransformed plants were treated similarly as controls. After 48 h, leaf nodes 1 to 3, 5, and 7, and stems at internodes 7 and 8 of treated and control (nontreated) plants were harvested for histochemical GUS staining and fluorometric GUS assay. Histochemical GUS staining was performed as described above with the following modification. Prior to incubation with GUS buffer, plant sections were incubated in 20% (v/v) methanol for 30 min. The GUS buffer contained 10 mM NaPO_4_ pH 7.0, 10 mM EDTA pH 8.0, 0.5 mM potassium ferricyanide, 0.5 mM potassium ferrocyanide, 0.1% (v/v) Triton X-100, 1% (v/v) DMSO, 20% (v/v) methanol and 0.5 mg/mL X-Gluc. Samples were incubated in GUS buffer for 14 h.

### 3.7. Protein isolation and fluorometric GUS assay

Leaf and stem tissues were weighed and frozen in liquid N_2_. Frozen tissues were ground to a fine powder with a pestle and extracted at a ratio of 300 mg fresh weight to 1 mL extraction buffer (50 mM NaPO_4_, pH 7.0, 10 mM β-mercaptoethanol, 10 mM EDTA, 0.1% (w/v) SDS and 0.1% (v/v) Triton X-100). The homogenates were centrifuged twice at 12,000 g for 15 min.

GUS activity was assayed fluorometrically by measuring the 456-nm emission from the 365-nm excitation. Leaf homogenates containing 5 µg total protein were added to 400 µL 2 mM 4-methylumbelliferyl β-D-glucuronide (4-MUG) and incubated for 0, 1 and 2 h at 37 °C. The reaction was stopped by adding 1.6 mL of 0.2 M sodium bicarbonate and the fluorescence was measured using 4-methylumbelliferone (4-MU) as a standard.

## 4. Conclusions 

PPO B, one of the seven PPO gene family members in tomato, was spatially and temporally regulated in vegetative and reproductive tissues under normal growth and differentiation and in response to ethylene. The 5’ flanking sequence of PPO B was partially homologous to sequences of genes encoding enzymes/proteins active in the following systems: phenylpropanoid biosynthesis, signal transduction and responsiveness to hormones and stresses, fruit and seed proteins/enzymes, and photosynthesis. Several putative *cis*-elements related to hormonal and environmental responses were identified in the PPO B promoter region. Promoter sequence homologies and PPO B-driven GUS expression patterns suggested a role for PPO B in programmed cell death and/or plant defense. However, these putative roles required further evaluation by functional analysis using RNAi to inhibit individual PPO B gene specifically. The knowledge will provide insight into how PPO could be manipulated to allow maximal benefits of reduced browning and enhanced pest resistance without deleterious effects on plant growth and development.
